# Effects of intestinal microbiota on physiological metabolism and pathogenicity of *Vibrio*

**DOI:** 10.3389/fmicb.2022.947767

**Published:** 2022-08-23

**Authors:** Han Sun, Changliang Zhu, Xiaodan Fu, Shakir Khattak, Jingyu Wang, Zhihao Liu, Qing Kong, Haijin Mou, Francesco Secundo

**Affiliations:** ^1^College of Food Science and Engineering, Ocean University of China, Qingdao, China; ^2^State Key Laboratory of Food Science and Technology, China-Canada Joint Laboratory of Food Science and Technology (Nanchang), Key Laboratory of Bioactive Polysaccharides of Jiangxi, Nanchang University, Nanchang, China; ^3^Istituto di Scienze e Tecnologie Chimiche “Giulio Natta”, CNR, Milan, Italy

**Keywords:** *Vibrio*, intestinal microflora, physiological metabolism, pathogenicity, colonization

## Abstract

*Vibrio* species are disseminated broadly in the marine environment. Some of them can cause severe gastroenteritis by contaminating seafood and drinking water, such as *Vibrio parahaemolyticus, Vibrio cholerae*, and *Vibrio vulnificus*. However, their pathogenic mechanism still needs to be revealed to prevent and reduce morbidity. This review comprehensively introduces and discusses the common pathogenic process of *Vibrio* including adhesion, cell colonization and proliferation, and resistance to host immunity. *Vibrio* usually produces pathogenic factors including hemolysin, type-III secretion system, and adhesion proteins. Quorum sensing, a cell molecular communication system between the bacterial cells, plays an important role in *Vibrio* intestinal invasion and colonization. The human immune system can limit the virulence of *Vibrio* or even kill the bacteria through different responses. The intestinal microbiota is a key component of the immune system, but information on its effects on physiological metabolism and pathogenicity of *Vibrio* is seldom available. In this review, the effects of intestinal microorganisms and their metabolites on the invasion and colonization of common pathogenic *Vibrio* and VBNC status cells are discussed, which is conducive to finding the next-generation prebiotics. The strategy of dietary intervention is discussed for food safety control. Finally, future perspectives are proposed to prevent *Vibrio* infection in aquaculture.

## Introduction

*Vibrio* species are disseminated broadly in aquatic environments, including estuaries, marine coastal waters and sediments, and aquaculture settings (Bonnin-Jusserand et al., 2019; Valente and Wan, 2021). *Vibrio cholerae, Vibrio parahaemolyticus*, and *Vibrio vulnificus* are the most common pathogenic species that contaminate seafood and drinking water causing heavy food poisoning incidents and serious illness in humans (Thorstenson and Ullrich, 2021). Cholera, a severe disease that is still occasionally prevalent in developing countries, is caused by *V. cholerae* and transmitted through polluted food and water because of inadequate sanitation of the food and water chain supply (Islam et al., 2004). The infection of *V. cholerae* remains a global health concern because of the over 100,000 deaths per year by O1 and O139 serogroups (Cho et al., 2021). In human hosts, *V. cholerae* preferentially colonize the duodenum, producing cholera toxin (CT) and the toxin-coregulated pilus (TCP) for colonization and cellular damage.

**Figure 1:**
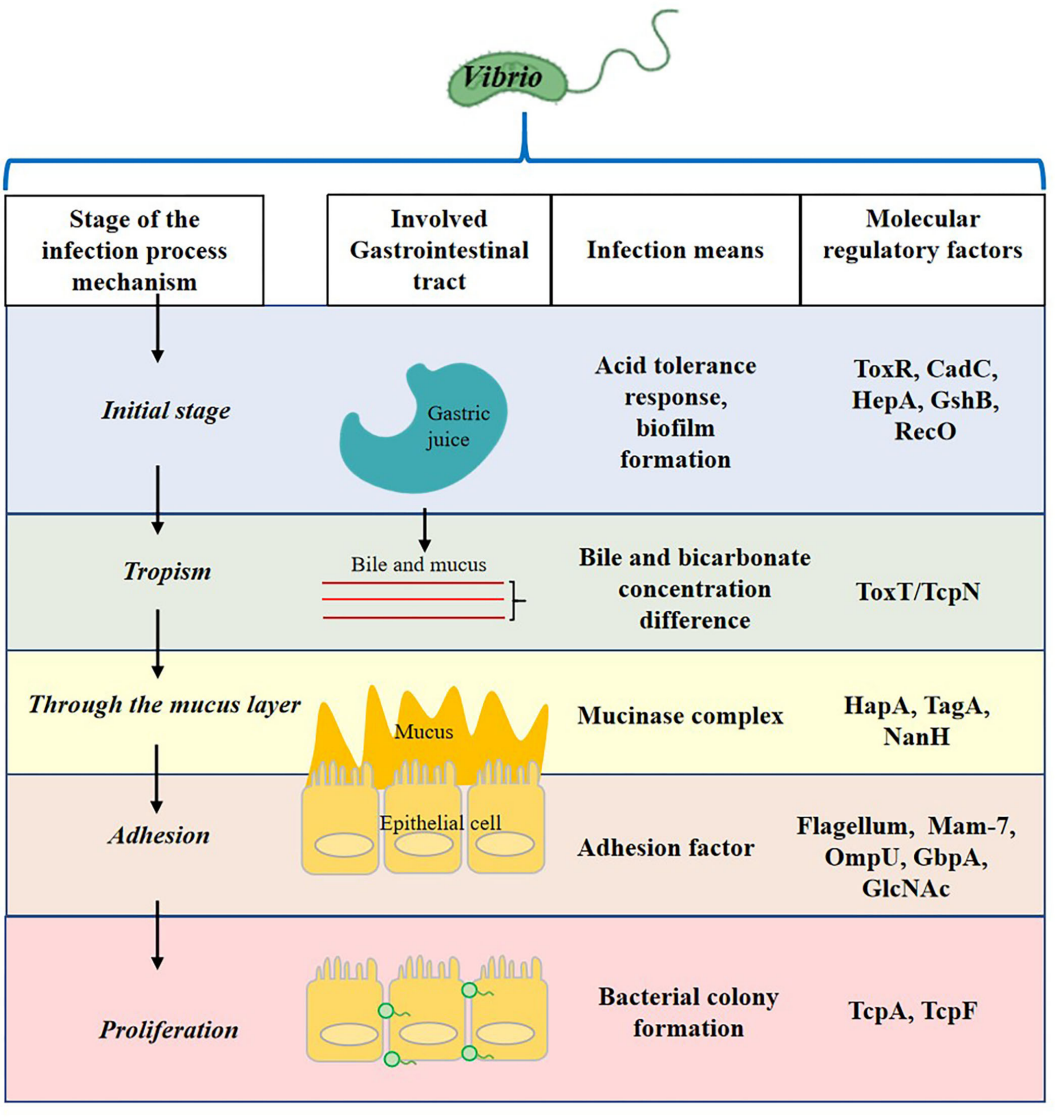
The involved gastrointestinal tract, infection means, and molecular regulatory factors of the *Vibrio* infection process.

*Vibrio* infection process contains the initial stage in the stomach, tropism to epithelial cell, through mucus layer, and adhesion and proliferation (Figure 1). After *Vibrio* invades the human intestinal tract, it produces complex and diverse pathogenic factors to ensure the infection of the host. These include thermostable direct hemolysin (TDH), TDH-related hemolysin (TRH), type III secretion system (T3SS), and adhesion proteins (Ritchie et al., 2012; Zhang and Kim, 2013), for example, the multivalent adhesion molecule (MAM) 7 by which the pathogen adheres to the epithelial lining of the small intestine during the early infection of *V. parahaemolyticus* (Krachler and Orth, 2011). In addition, the infected cells will face complex microecological relationships in the intestinal environment, which seriously affects the growth and colonization of *Vibrio*. Among them, quorum sensing (QS), the cell-to-cell communication system implemented by signaling molecules (autoinducer) and based on population density, may play one of the most important roles in intestinal colonization and invasion (Gode-Potratz and McCarter, 2011). It is found that QS can regulate adhesion factors of *Vibrio*, such as T3SS, MAM, and flagella. A previous study revealed the attachment of *V. parahaemolyticus* to mammalian cells was reduced from 80 to 35% in the absence of MAM-7 (Krachler and Orth, 2011). Therefore, it is significant to find concrete evidence which shows an association between QS and bacterial virulence for *Vibrio* (Zhang et al., 2012b; Bonnin-Jusserand et al., 2019).

To maintain the balance of intestinal microecology, native intestinal microorganisms will also inhibit and reject the invading microbial cells. Intestinal contents, including intestinal microbial metabolites and tissue secretions, may affect a series of physiological and growth behaviors of *Vibrio*, which may be completely different from that of pure culture *in vitro*, especially concerning the expression level of factors related to cell invasion and pathogenicity. The previous study injected *V. vulnificus* K44 or R41 into mice and found although the two strains induce macrophage apoptosis *in vitro*, only K44 could escape from the host defense *in vivo* (Kashimoto et al., 2003). In addition, *V. harveyi* autoinducer 1 (HAI-1) is the channel of QS. The *in vivo* experiment revealed it was inactive during infection of brine shrimp, which might suggest this signal had low stability or was not produced *in vivo* (Defoirdt et al., 2008).

When bacteria are exposed to some environmental stresses, such as low temperature and nutrition starvation, they enter an unusual physiological state in which they cannot grow on common culture media but still perform their cellular activities called viable but non-culturable (VBNC). Some *Vibrio* species, including *V. cholerae*, became a special survival form of bacteria (Ayrapetyan and Oliver, 2016). The pathogens, when in the VBNC state, are usually unable to cause disease, but in the human gastrointestinal tract they can restart growing, regain their pathogenic properties, and cause severe illness (Nicolò and Guglielmino, 2012). Thus, the interaction of *Vibrio* with intestinal microorganisms can trigger the restarting of *Vibrio* growth.

By now, little is known about the effects of intestinal microorganisms on the physiological metabolism and pathogenicity of *Vibrio*. In the current review, we focus on the effects of intestinal microbiota and their metabolites on the invasion and colonization ability of common pathogenic *Vibrio* species. The key gene expression of *Vibrio* including virulence and adhesion factors and the effects of microbiota on the restarting of VBNC state *Vibrio* are also discussed. Besides, this review aims to provide some suggestions for dietary intervention for food safety control involving *Vibrio* contamination and other pathogens.

## Pathogenic mechanism of common pathogenic *Vibrio* species

The *Vibrio* pathogenic process usually includes adhesion of the pathogen to the intestinal mucus layer, invasion of host tissue, colonization and cell proliferation, and resistance to the host immunity, but *V. cholerae* is not invasive. During the process, *Vibrio* cells produce different types of toxins important for the virulence of the pathogen.

During adhesion to the host cell, the TCP, a type IV bundle-forming pilus, is the main colonization factor for the pathogenicity of *V. cholerae*. It is a polymer of subunits of TcpA, whose C-terminal region is exposed on the surface of pilus fiber to regulate the colonization functions of TCP. The TcpA was expressed in tomatoes to be an antigen for anti-colonization immunity (Sharma et al., 2008). The GbpA is the N-acetylglucosamine-binding protein in *V. cholerae*, participating in *V. cholerae* attachment. It has a four-domain structure, and three of them are involved in the colonization process (Wong et al., 2012). Other adhesion factors such as OmpU and MAM7 are also identified in *Vibrio* species (including non-cholerae ones) and their role depends on the context of the pathogenic process (Liu et al., 2018; Crisan and Hammer, 2020; Ganie et al., 2022). The MAM7, T3SS, and T6SS are the main factors associated with *V. cholerae, V. parahaemolyticus*, and *Vibrio alginolyticus*, and T3SS has a little correlation with *V. vulnificus* (Table 1). After the adhesion, certain *Vibrio*, including *V. cholerae, V. parahaemolyticus, V. vulnificus, Vibrio harveyi*, and *V. alginolyticus*, starts the biofilm formation that is the main step toward the disease development (Ashrafudoulla et al., 2020). In these conditions, gene expression at the stationary phase is regulated by bacterial cell-to-cell communication. The LuxR or its homolog is detected in all studied *Vibrio* to date and is considered to regulate *cps* gene expression positively for biofilm formation. Thus, the mature biofilm exhibits a gene expression pattern, which is beneficial for the resistance to environmental stressors (Wang et al., 2011). However, colonization of *V. cholerae* in the small intestine is negatively affected by the inhibition of motility, which also decreases biofilm formation *in vitro* (Purdy and Watnick, 2011). Other studies also indicate that biofilm formation of *V. cholerae* is critical for intestinal colonization (Silva and Benitez, 2016), but information on its *in vivo* formation is seldom available. The biofilm gene of *vps* was expressed and essential for intestinal colonization of *V. cholerae* O139 in *Drosophila melanogaster* (Blow et al., 2005). However, the mechanism by which flagellar movement affects surface adhesion *in vivo* is not fully understood. Silva and Benitez (2016) suggest that adhesion can be regulated by flagellar movement and sodium flux of membrane potential. The synergistic effect of flagella and mannose-sensitive hemagglutinin (MSHA) type IV pili of *V. cholerae* allows a surface interaction to enhance the adhesion (Utada et al., 2014). However, it is difficult to confirm such a mechanism in the detail because of the complex flagellar state *in vivo*.

**Table 1:** Different virulence factors of *Vibrio* species during the infection process.

***Vibrio*/Virulence** **factors**	***V. cholerae***	***V. parahaemolyticus***	***V. vulnificus***	***V. alginolyticus***	**References**
Adhesion	MAM7, T3SS (except for O1/O139 ones), T6SS, TCP, GbpA	MAM7, T3SS, T6SS	MAM7, T6SS	MAM7, T3SS, T6SS	Wong et al. (2012), Church et al. (2016)
Hemolysin	CT	TDH, TRH, TLH	VvhA	TDH, TRH, TLH	Church et al. (2016)
Enzymatic reactions	Urease, lipase, gelatinase	Urease, lipase, gelatinase	Urease, lipase, gelatinase	Lipase, gelatinase	Baffone et al. (2001)

The Gram-negative bacteria contain secretory systems that are essential for the invasion. Different secretory systems have been reported in *Vibrio* including T2SS, T3SS, and T6SS (Zhao et al., 2018). Among them, T2SS of *V. vulnificus* causes lysis and necrosis of epithelial cells (Jang et al., 2017). By examining 110 *Vibrio* strains, T3SS1 was detected in *V. parahaemolyticus, V. alginolyticus, V. harveyi*, and *V. campbellii*, but T3SS2 was only found in *V. parahaemolyticus* RIMD2210633 and ATCC33847 (Wu et al., 2020). T3SS2 was reported to be functional in a few *V. parahaemolyticus* and *V. cholerae* strains (Miller et al., 2019). T3SS1 mainly affects the biofilm formation, motility, and cytotoxicity and contributes to the survival of *Vibrio* in the environment, whereas T3SS2 of *V. parahaemolyticus* is involved in the negative regulation of the cellular inflammatory response, which is conducive to the process of evasion of host immune system by the pathogenic bacteria (Park et al., 2004; Calder et al., 2014). T3SS was reported to be mainly responsible for diarrhea caused by non-O1/O139 *V. cholerae* but played no role at all in O1/O139 ones (Shin et al., 2011). The *V. parahaemolyticus* that lack T3SS2 are unable to colonize the intestinal environment of infant rabbits and do not develop the disease symptoms in ligated ileal loops (Ritchie et al., 2012). Further study suggested that T3SS2-lacking *V. parahaemolyticus* could colonize the intestinal lumen of germ-free mice but it did not cause an obvious inflammatory response (Yang et al., 2019). T6SS, mainly studied in *V. cholerae*, participates in the adhesion to cultured cell monolayers and injects virulence factors (Ye et al., 2008). T6SS1 is most active under warm marine-like conditions (Salomon et al., 2013), and T6SS2 is active under low salt conditions (Yu et al., 2012).

During the pathogenic process, toxins produced by *Vibrio* depend on the context, that is, on the species and environments (Table 1). They cause severe symptoms like fever, watery and bloody diarrhea, and vomiting. The CT is the main virulence factor of *V. cholerae* infections encoded by *ctx*A and *ctx*B (Bonnin-Jusserand et al., 2019). *Vibrio parahaemolyticus* causes enterotoxicity and cytotoxicity due to TDH, TRH, and thermolabile hemolysin (TLH). *Vibrio alginolyticus* also contains the TDH and TRH like those found in *V. parahaemolyticus* (Cai et al., 2007). In *Vibrio vulnificus*, the *vvhA* gene encodes the toxin protein having hemolytic activity able to not only destroy the host red blood cells and release iron for the use of bacteria but also cause a strong cytotoxic effect that evolves into serious tissue necrosis (Chung et al., 2016). Many *Vibrio* species including *V. cholerae, V. parahaemolyticus, V. vulnificus, and V. alginolyticus* contain the *toxR* gene, which is an ancestral locus in *Vibrio* (Chen et al., 2012). Other virulence factors, such as urease and lipase, also affect the transcription of virulence genes in various *Vibrio* species (Baffone et al., 2001).

The colonization of *Vibrio* occurs by the effect of physical adhesion factors and after the antagonistic interaction between the pathogen with intestinal bacteria. It was proved that TDH facilitates the colonization of *V. parahaemolyticus* and *V. alginolyticus* by damaging the intestinal epithelial cells leading to the colonization of bacteria in a large number. Metalloprotease VvpE in *V. vulnificus* disrupts the intestinal wall by interacting with the intestinal proteins responsible for bacterial pathogenesis and promotes intestinal colonization of the pathogen (Lee et al., 2016).

The QS is cell-to-cell communication by which bacteria coordinate with each other based on cell density (Hawver et al., 2016). During resistance to the host immunity, *V. cholerae* has the efficient QS mechanism by which the cells communicate to produce pathogenic factors participating in hemolysis, biofilm formation, secretion systems, metabolic fitness, and swarming (Suckow et al., 2011; Shao and Bassler, 2014; Jemielita et al., 2018). QS regulates virulence factors of *Vibrio* through different bacterial densities. For example, at OD_600_ values of 0.05 to 0.2, AphA activated the expression of VPA0606 in *V. parahaemolyticus* to promote biofilm formation and increase bacterial mobility and cytotoxicity. At an OD_600_ value of 0.2 to 0.4, the transcriptional expression of ToxR is indirectly inhibited by AphA, resulting in reduced cytotoxicity (Zhang et al., 2017). It is known that QS regulates (i) ToxR expression that activates the expression of T3SS and T6SS (Li et al., 2019), (ii) the lateral flagellum genes that influence the movement and colonization of *V. parahaemolyticus* (Lu et al., 2019), (iii) LuxS that upregulates biofilm formation by stimulating flagella formation and exopolysaccharides production in *V. alginolyticus* (Ye et al., 2008). SmcR is the QS master regulator in *V. vulnificus*, which regulates the expression of hemolysin and elastase (Lee et al., 2007). The direct binding of the agent of antivirulence to SmcR was proved to effectively alleviate the virulence of *V. vulnificus* (Kim et al., 2020). QS and ToxS, as the centers of regulation, can regulate the genes involved in biofilm formation and those related to pathogenic factors to influence the virulence of *V. cholerae, V. parahaemolyticus*, and *V. alginolyticus*. In addition, cyclic diguanylate (c-di-GMP) is an allosteric activator that regulates the transition between sessility and motility in *V. cholerae* and *V. vulnificus* (Srivastava et al., 2014; Park et al., 2015). In turn, the intracellular concentration of c-di-GMP is regulated by σ^54^-dependent activator FlrA, biofilm activators VpsR and VpsT (Silva and Benitez, 2016). The elevated c-di-GMP level was reported to promote biofilm formation by *V. vulnificus* (Park et al., 2015). In *V. cholerae*, the VieA was reported to control c-di-GMP concentration regulating exopolysaccharide synthesis involved in biofilm formation (Tischler and Camilli, 2004).

## Effects of intestinal microbiota on colonization and invasion of pathogenic *Vibrio*

**Figure 2:**
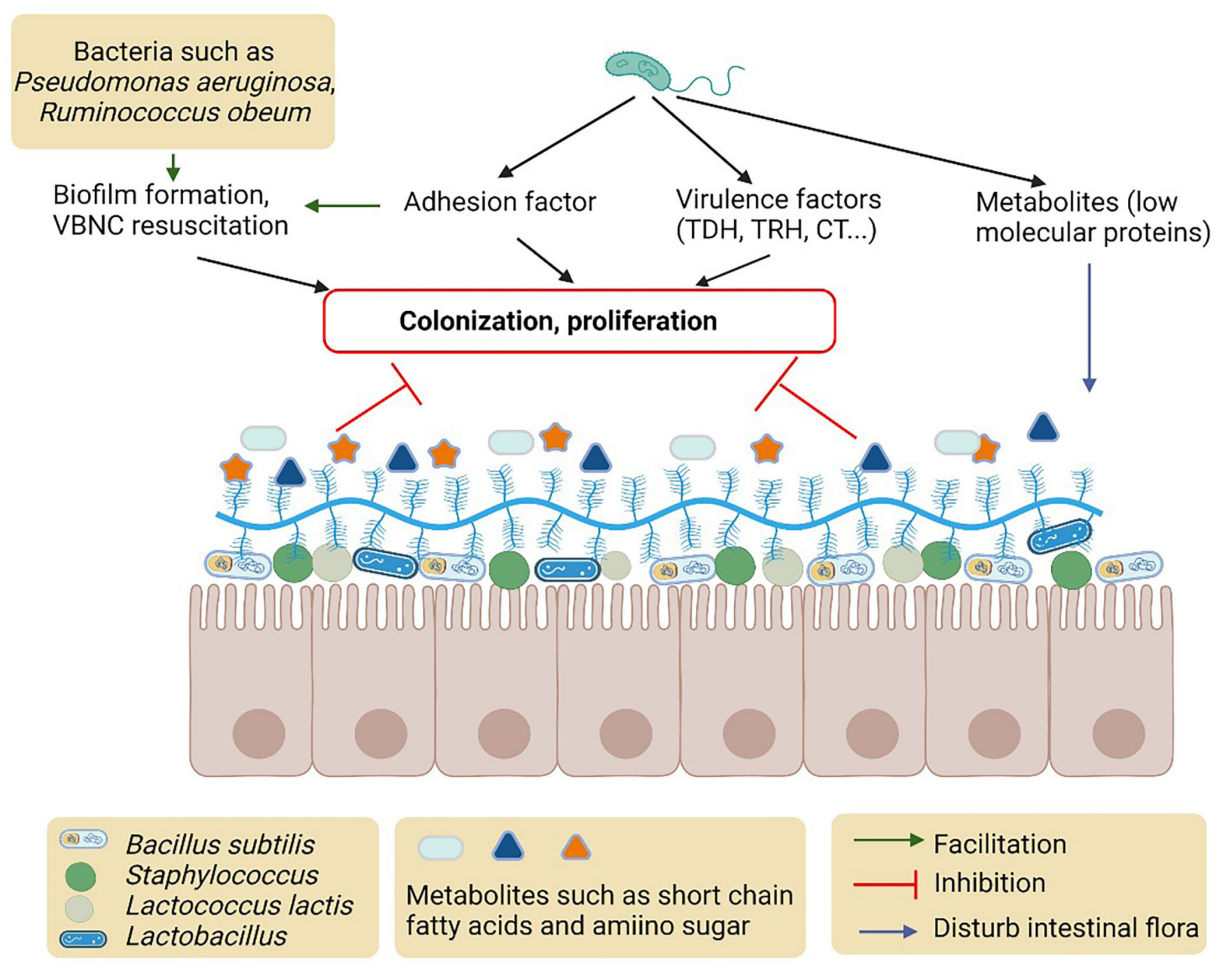
Possible mechanisms and metabolites of intestinal microbiota to inhibit colonization of proliferation of *Vibrio*.

There are many factors produced by intestinal normal microbiota (e.g., *Escherichia coli*), opportunistic pathogens (e.g., *Pseudomonas aeruginosa* and *Bacteroides fragile*), and probiotics in the intestinal tract that interfere with *Vibrio* pathogenesis (Gopalakrishnan et al., 2015; He et al., 2017) (Figure 2). Intestinal microbiota plays an important role in the regulation of virulence gene expression including MAM7, TDH, TRH, biofilm, and T6SS in *Vibrio* (Zhang et al., 2012a). The colonization of *V. parahaemolyticus* is influenced by the sigma factor of *E. coli* in the human intestinal tract (Yu et al., 2012). *Bacteroides fragile* protects the macrophages in the intestine and its incorporation into the intestinal epithelial cells also inhibits *V. parahaemolyticus* colonization (Li et al., 2017b). The metabolites, including short-chain fatty acids and amino sugars, of intestinal microbiota, were also reported to protect the epithelial cell from colonization and invasion of *V. cholerae* (Qin et al., 2020).

It is observed that the heterogeneity of the intestinal microbial flora can significantly inhibit the invasion of the *Vibrio*. The colonization and invasion of *Vibrio* in the human intestine develop some clinical manifestations including diarrhea, vomiting, and high fever. After *Vibrio* infection, the intestinal wall will be damaged by the synthesis and secretion of toxic metabolites, and the structure of intestinal flora will be harmful to human health. *V. cholerae* infected the intestinal flora, while gut bacteria influenced the invasion of the pathogen. In mice, after eliminating much of the mouse gut microbiota by antibiotic treatment for 2 days, the immune response of host cells was reduced due to the change of heterogeneity of the intestinal microbial flora (Zhao et al., 2018). It was found that the microbiota possibly prevents infection of the invading pathogen by activating the host cell defense, competition for nutrients, or sites of adherence (Fukuda et al., 2011; Kamada et al., 2012). Competition for different nutrients by the intestinal normal flora acts as a barrier for the enteric pathogen. Metabolic competition for carbon sources, trace metals, and vitamins by the pathogenic bacteria and microbiota cause colonization resistance. Gut microbial can regulate microbial ecology by absorbing scarce vitamin B12 and inhibiting the growth of pathogens (Degnan et al., 2014).

Intestinal microbiota may also provoke *V. cholerae* by affecting the expression of T6SS (Zhao et al., 2018). This antagonism brings significant changes in the *V. cholerae* pathogenicity by enhancing MAM7, T6SS expression, and intestinal colonization. The antagonistic effect of *V. cholerae* on the intestinal microbiota can improve the pathogen's fitness which can make it easy for the pathogen to infect a new host (Kumar et al., 2020). The antagonism of *V. cholerae* may be related to the struggle of bacteria for nutrients, citrate utilization, and gluconeogenic action (Gopalakrishnan et al., 2015; Wang et al., 2018). After *Vibrio* enters the intestine, it inevitably competes with the intestinal microorganisms and even inhibits their growth through the synthesis of metabolites, thus interfering with the original intestinal microecological balance. The metabolites produced by *Vibrio* have a significant impact on intestinal microbiota. For example, *V. alginolyticus* primary metabolites may restrict *Staphylococcus aureus* to grow (Nursyam, 2017).

## Effects of QS mediated by intestinal microbiota on *Vibrio*

Intestinal microbiota also mediates the QS to regulate adhesion, colonization, and invasion of *Vibrio* (Hsiao et al., 2014) (Figure 2). *N*-hexanoyl-l-homoserine lactone (AHL) of *Pseudomonas aeruginosa* affects the biofilm formation of *V. parahaemolyticus* in zebrafish. *Pseudomonas aeruginosa* increases the enzymatic activity of superoxide dismutase and lysozyme, the biofilm polysaccharides organization, and helps raise the phagocytic cell of the host immune system (Gopalakrishnan et al., 2015). *Vibrio cholerae* signaling factor interferes with the QS system of *P. aeruginosa* influencing the physiological conditions of intestinal microbiota. Upon *V. cholerae* infection, *Ruminococcus obeum* exhibits a consistent increase in its relative abundance. *Ruminococcus obeum* luxS (AI-2) expression increases significantly with *V. cholerae* invasion and causes QS-mediated repression of *V. cholerae* colonization factors. *Ruminococcus obeum* AI-2 reduces *V. cholerae* colonization and pathogenicity through a novel pathway, LuxP (Hsiao et al., 2014). Furthermore, it has been found that some strains of *V. alginolyticus* produce QS inhibitors that cause the stoppage of gut microbiota phenotypes of *P. aeruginosa* (Reina et al., 2019).

## Effects of intestinal microorganisms and their metabolites on VBNC state *Vibrio*

Viable but nonculturable (VBNC) is the state in which the bacteria are unable to grow on the culture media but are still alive. It is considered an adaptive strategy for survival in *Vibrio* in which the pathogen escapes the unfavorable environment. There are different stress conditions including drying, oxidative stress, radiation disinfection, and antiseptic that cause the VBNC state of the pathogen. At the oxidative stress condition, *V. vulnificus* ATCC27562 becomes non-culturable (Abe et al., 2006). *Vibrio cholerae* O1 El Tor strain adopts the VBNC state at 4°C in artificial seawater within 35 days. *Vibrio alginolyticus* also adopts the VBNC state in the same conditions (Albertini et al., 2010; Xu et al., 2018).

The restarting from the VBNC state into the culturable state is possible when the environmental conditions are favorable, and the bacteria can perform all the normal metabolic activities. Currently, many strategies are tested for the restarting of VBNC bacteria. High temperature (Du et al., 2007), the addition of chemical substances into the environment (Mizunoe et al., 2000), and co-culture with host cells (Senoh et al., 2010) allowed the restarting of *V. cholerae* O1/O139 and *V. parahaemolyticus* 23552 from the VBNC state. The bacteria in the VBNC state co-cultured with Chinese hamster ovary cells stimulate the pathogen to recover its normal biological form.

Although there are still many problems to be understood about the recovery mechanism of VBNC bacteria, it has been found that many microbial metabolites play an important role. For example, some pathogens, such as *E. coli* O157:H7, can recover their active form using their autoinducers produced during biofilm formation (Liu et al., 2009). It is suggested that the metabolic molecule of pyruvate in the medium during the bacterial growth may promote the conversion of culturable to VBNC state (Morishige et al., 2013). However, the metabolic products that act as resuscitative agents remain unknown. Sodium pyruvate may be the key molecule in the resuscitation process of *Salmonella enteritidis*, which restores the biosynthesis of macromolecules such as DNA and proteins, thereby converting the VBNC cells to a culturable state (Mukamolova et al., 2002). Recently, a recovery-promoting factor (Rpf), as a secretory protein of *Micrococcus luteus* (Arana et al., 2004), was proved to participate in the restarting of VBNC cells, which may have either autocrine (by the same cells that produce them) or paracrine (may act on the nearby cells) signaling functions.

So far, good progress has been made in the research on the reactivation of VBNC bacteria entering the human intestine, but it remains unclear the role of intestinal microbiota and their metabolites, and the host-bacterial interactions (Li et al., 2014). Therefore, studies that shed light on the effects of intestinal microorganisms and their metabolites on the reactivation of the VBNC state *Vibrio* are very desirable.

## Dietary intervention on *Vibrio* pathogenicity by regulating the intestinal microbiota

### Probiotics and phages

The use of antibiotics and chemotherapeutic agents helps to control infections in the breeding industry, but they also cause the development of drug-resistant bacteria and food-safety related problems (Giri et al., 2015; Zhang et al., 2020b). Probiotics are considered a sustainable and promising solution because they are environment-friendly and help host growth, immunity, and disease control (Lazado and Caipang, 2014). Therefore, the use of probiotics in treating diseases of livestock is increasing. The consumption of probiotics and their functionally active substances improve the reasonable gut microbiota structure, which may help in the inhibition of the invasion of *Vibrio* (Table 2). The harmful effects of *V. parahaemolyticus* in mice can be minimized by taking probiotics such as *Streptomyces* and *Lactobacillus* in *Litopenaeus vannamei*, which enhance the intestinal microbiota (Reina et al., 2019). Also, the adhesion of *V. parahaemolyticus* can be lowered by the intake of probiotics (Satish Kumar et al., 2011). A significant reduction of *Vibrio* spp. was observed in the Pacific white shrimp after feeding it with microencapsulated probiotics, *Bacillus subtilis*, or *Staphylococcus lactis* (Boonanuntanasarn et al., 2016). The shrimp diet supplemented with *Lactococcus lactis* can increase resistance to *V. alginolyticus* and increase *Lactobacillus* and *Bacillus* count in the shrimp intestine and reduces the number of *Vibrio* species (Adel et al., 2017). The number of intestinal bacteria can be increased by *Halomonas* B12 which causes a significant reduction in *Vibrio* spp. bacterial count in Chinese shrimp (Zhang et al., 2009).

**Table 2:** Dietary intervention on *Vibrio* pathogenicity by regulating the intestinal microbiota.

**Category**	**Substance**	**Function**	**Reference**
Probiotics and phages	*Streptomyces* and *Lactobacillus*	Reduction of the harm of *Vibrio parahaemolyticus*	Reina et al. (2019)
	*Lactobacillus plantarum*	Inhibition of adhesion of *Vibrio parahaemolyticus*	Satish Kumar et al. (2011)
	*Bacillus subtilis* or *Staphylococcus lactis*	Significant reduction of *Vibrio* in the Pacific white shrimp	Boonanuntanasarn et al. (2016)
	*Lactococcus lactis*	Increase resistance toward *V. alginolyticus*	Adel et al. (2017)
	*Halomonas B12*	Cause significant reduction in *Vibrio* bacterial count in Chinese shrimp	Zhang et al. (2009)
	*Lactobacillus*	Boost the immunity resistance to *V. parahaemolyticus*	Elshopakey et al. (2018)
	*Lactobacillus lactis*	Inhibit the biofilm formation of *V. cholerae*	Kaur et al. (2018)
	*Escherichia coli 40* and Nissle 1917	Reduce pH value in the medium and affect the survival rate of *V. cholerae*	Sengupta et al. (2017)
	*Ruminococcus obeum*	Reduce *V. cholerae* colonization and pathogenicity	Hsiao et al. (2014)
	Three phages, PVA1, PVc1 and PVS3	*Vibrio* in sea cucumber can be reduced by mixing different phage mixtures	Li et al. (2020)
Carbohydrates	Sialyl-3′-lactose; sialyl-3P-lacto-*N*-neotetraose	Anti-adhesion of pathogens	Ofek et al. (2003)
	Mannan oligosaccharide	Causes the reduction of *Vibrio* and improve the intestinal microbiota	Dimitroglou et al. (2009)
	Xylose oligosaccharide	Increase survival rate of *L. vannamei* against *V. alginolyticus*	Sun et al. (2019)
	Chitosan	Increase survival rate of mice after infection with *V. vulnificus*	Lee et al. (2009)
	Isomalto-oligosaccharides	Lower mortality in the experimental shrimps infected with *V. alginolyticus*	Zhang et al. (2011)
	Pectin oligosaccharides	Anti-adhesion of *V. cholerae*	Wang et al. (2015)
	Fucosylated oligosaccharides Sialic acid oligosaccharides	Inhibit *V. cholerae* ATCC14034 adhesion to Caco-2	Coppa et al. (2006)
Proteins	By-product meal and fish meal	Improve the resistance toward pathogens	Siddik et al. (2019a)
	Fish meal	Increase resistance toward *V. anguillarum*	Torrecillas et al. (2017)
	Tuna hydrolysate	Increase resistance against *V. harveyi* infection in fish	Siddik et al. (2019b)
	Peptides	Restrict *Vibrio* growth and increase host resistance vs. *V. parahaemolyticus*	Liao et al. (2019)
	Glycinin	Reduces the *Vibrio* count	Li et al. (2017a)
	Soybean meal	Reduce number of *Vibrio* species	Dimitroglou et al. (2009)
Lipids and organic acids	Oregano essential oil	Prevent from infections, enhances the resistance of animals to *Vibrio*	Gracia-Valenzuela et al. (2014), Zhang et al. (2020a)
	Short-chain esters	Reduce number of *V. cholerae*	Petschow et al. (1998)
	Gelatinized polyhydroxy butyrate	*Penaeus vannamei* acquired 100% survival rate against *Vibrio*	Kiran et al. (2020)
	Sunflower oil	Reduce mortality of Atlantic salmon	Bransden et al. (2003)
	Thymol and carvacrol	Change the intestinal microbiota of tilapia	Ran et al. (2016)
	Organic acids, sodium propionate, citric acid, essential oils	Inhibit the *Vibrio* growth and enhance the intestinal microbiota	da Silva et al. (2016), Chen et al. (2018), Wassef et al. (2020)
	cider vinegar, propionic acid	Reduce *Vibrio* count significantly	Pourmozaffar et al. (2019)

The types of microecological agents used to control intestinal microbiota are expanding. The addition of yeast and fermented vegetable product by *Lactobacillus* to the diet of Kuruma shrimp can boost the immunity resistance to *V. parahaemolyticus* (Elshopakey et al., 2018). In sea cucumber, *V. alginolyticus, V. splendidus, and V. cyclitrophicus* can be reduced by mixing different phage mixtures in a certain proportion, but in the experimental group treated with antibiotics, the number of *Vibrio* has not changed significantly (Li et al., 2020).

### Carbohydrates

Diet plays an important role in the regulation of the intestinal microbiota (Krachler and Orth, 2011). Diarrheal disease is mainly treated through oral rehydration therapy comprising glucose. However, the virulence gene expression and toxin production were enhanced in a glucose-concentration manner. Instead, the rice-based oral rehydration therapy decreased the virulence determinants (Kühn et al., 2014). In addition, it is widely accepted that intestinal microbiota can be improved by dietary prebiotics (Boonanuntanasarn et al., 2016), and some oligosaccharides are effective in the anti-adhesion of pathogens *in vitro* and *in vivo* studies (Ofek et al., 2003). For example, the intestinal microbiota is improved by mannan oligosaccharide (MOS) in trout and causes the reduction of *Vibrio* (Dimitroglou et al., 2009). The *L. vannamei* fed with xylose oligosaccharide shows a significantly increase survival rate against the challenge of *V. alginolyticus* (Sun et al., 2019). Similarly, mice fed with chitosan show a higher survival rate than the control after infection with *V. vulnificus* (Lee et al., 2009).

It was reported that in the shrimp intestine, the bacterial count of normal gut microbiota significantly increased and the bacterial count of *V. alginolyticus* decreased with symbiotic supplemented dietary. *Bacillus licheniformis* and *B. subtilis* in the presence of isomalto-oligosaccharides in the feed increase resistance to *V. alginolyticus*, showing lower mortality in shrimps infected with *V. alginolyticus* compared to the control group (Zhang et al., 2011).

*Vibrio cholerae* adhesion to human intestinal epithelial cell line HT-29 can be significantly inhibited by pectin oligosaccharides (Wang et al., 2015). Human milk containing fucosylated oligosaccharides and sialic acid oligosaccharides can inhibit *V. cholerae* ATCC14034 adhesion to Caco-2 (Coppa et al., 2006). Beyond oligosaccharides, bacterial adhesion can also be inhibited by some monosaccharides (Wang et al., 2015).

### Proteins

*Vibrio* is a very common pathogenic bacteria in aquaculture animals, and the use of vegetable protein raw materials, such as soybean meal, to replace fish meal has become one of the most important measures to reduce the cost of the industry. Therefore, the impact of different types of protein raw materials on intestinal microorganisms of aquacultural animals and the pathogenicity of *Vibrio* has become common concerns of the aquaculture industry. It is most common to regulate the intestinal microbiota of animals by diet in the breeding industry. Moreover, some studies also suggested that enrichment of diet with fermentation, probiotics, and trace elements can improve the resistance to pathogens by strengthening the immune system of aquatic animals (Siddik et al., 2019a). Resistance of European seabass to *V. anguillarum* was increased with a fish feeding prepared with fish oil and fish meal mixed in different proportions by enhancing the intestinal microbiota (Torrecillas et al., 2017). Previous studies found that the *Aeromonas* count was significantly reduced and *Lactobacillus* and *Streptococcus* were enhanced by adding fermented poultry by-product meal to fish meal, while the survival rate of the fish was increased after *V. mimicus* infection (Siddik et al., 2019a). The addition of tuna hydrolysate to the diet of juvenile *Barramundi* can increase resistance against *V. harveyi* infection in fish (Siddik et al., 2019b) and adding peptides also restricts *Vibrio* growth and increase host resistance to *V. parahaemolyticus* (Liao et al., 2019). Studies show that glycinin does not affect the normal microbial community of the fish intestine after feeding, but it significantly reduces the *Vibrio* count (Li et al., 2017a). Rainbow trout fed with soybean meal for 16 weeks have a low number of *Vibrio* spp. in the gut compared to those fed with the fish meal (Dimitroglou et al., 2009).

### Lipids and organic acids

Lipid is the main component of food and an important energy source providing essential fatty acids for the body. In the current situation which bans antibiotics, plant essential oil attracts the interest of the aquaculture industry because of its significant bactericidal effect *in vitro*. Previous studies show that oregano essential oil can be used as a substitute for antibiotics because of its antibacterial activity, helping the body to prevent infections (Zhang et al., 2020a). It improves the intestinal bacteria of the animals and enhances the resistance of animals to *Vibrio*, the reduction of *Vibrio*, and increase of genera *Propionibacterium, Brevinema*, and *Cotynebacterium* were observed in the intestine of fish (Zhang et al., 2020a). The growth of *V. vulnificus, V. parahaemolyticus*, and *V. cholerae* can be significantly reduced in shrimp fed with oregano essential oil (Gracia-Valenzuela et al., 2014).

The antibacterial and intestinal repairing properties of some short-chain esters have also been observed. A study shows that the growth of *V. cholerae* ATCC25870 in the gut of Streptomycin-treated mice fed with monoglyceride was 1,000 times reduced in comparison with those fed with a normal diet (Petschow et al., 1998). The shrimp *Penaeus vannamei* fed with gelatinized polyhydroxy butyrate for 60 days acquired a 100% survival rate when infected with *V. parahaemolyticus* (Kiran et al., 2020).

Besides, high content of n-6 polyunsaturated fatty acids in the diet can help to strengthen body immunity. A notable reduction is a mortality of *Atlantic salmon* against the infection of *V. anguillarum* when sunflower oil was given in the diet (Bransden et al., 2003). Fat-soluble small molecules have also been shown to improve intestinal microbiota. Thymol and carvacrol have antibacterial properties when mixed in a certain ratio and can change the intestinal microbiota of tilapia (Ran et al., 2016).

To minimize the use of antibiotics and to improve the health in aquaculture, the aquafeed is supplemented with different organic acids (Wassef et al., 2020). The addition of organic acid to the diet can inhibit the growth of *V. cholerae, V. harveyi, V. parahaemolyticus, V. vulnificus, V. alginolyticus*, and *V. campbellii* and enhance the intestinal microbiota (da Silva et al., 2016). For example, the *Vibrio* spp. count significantly decreased when the diet was supplemented with apple cider vinegar and propionic acid (Pourmozaffar et al., 2019). After consuming organic acids and essential oils, the intestinal microbiota can diversify and be enriched in *P. vannamei*. Also, it was observed that the *Lactobacillus* growth was promoted and the resistance of shrimps to *V. parahaemolyticus* increased (He et al., 2017). The intestinal flora of the European seabass was enhanced when fed with sodium propionate, accompanied by a significant reduction of *Vibrio* spp. (Wassef et al., 2020). The consumption of citric acid also results in the reduction of *Vibrio* spp. in the Turbot gut (Chen et al., 2018).

Dietary intervention is a promising strategy to reduce the *Vibrio* pathogenicity in aquaculture. The antibiotic resistance of *Vibrio* impels the development of probiotics, phages, and other active compounds for reducing high morbidity. Such dietary intervention is usually involved in *Vibrio* colonization, nutrient competition, and virulence gene expression by regulating the intestinal microbiota. However, the *Vibrio* species in aquatic environments are different, and the intervention mechanism is discriminatory according to the aquatic livestock. Therefore, the rational design of dietary intervention is required based on the clear pathogenic mechanism of *Vibrio* species on specific livestock. The reagent dosage and safety are other factors to consider for the economic feasibility of aquaculture.

## Challenges and future perspectives

Nowadays, the mechanism of *Vibrio* pathogenicity has been explored by controlling the environmental variables, including temperature, salinity, and host organisms. However, several underlying mechanisms, such as biofilm formation and propagation mode worldwide, remain unclear, which is possibly due to the specificity of different *Vibrio* species during the pathogenic process. They differ in colonization and toxicity to the same host and environment. Therefore, network analysis and database establishment are beneficial to extend the technology application for preventing *Vibrio* in aquaculture. In addition, *Vibrio* pathogenicity to host organisms is a dynamic process involving energy and substance metabolism networks. To accurately elucidate the pathogenic mechanism, the combination of metabolic tools (such as multi-omics analysis) and experimental animal models is a promising systematic strategy. The multi-omics analysis including transcriptomics, metabolomics, and proteomics can provide the information involved in molecular mechanisms during the pathogenic process.

Besides, too many scientific problems including the exact mechanism by which the intestinal commensal bacteria interfere with the virulence gene expression are not well understood. To better control intestinal diseases caused by *Vibrio* and ensure intestinal health and food safety, studies that shed light on the complex mechanism of *Vibrio* after entering the intestine must be pursued. The new models of human gut microbiomes with *Vibrio* are needed to find the candidate probiotics. The oral vaccines or probiotics exhibit different efficacies in human populations, which are mainly ascribed to the gut microbiome. The probiotics can be applied in aquaculture and the food industry to improve food safety by enhancing the host defense system. Finally, once the infection occurs in aquaculture, the efficient detection of *Vibrio* species is significant for alleviating the infection spread to reduce economic losses. Therefore, the development of quick and easy-to-operate detection methods is essential in future studies.

## Conclusion

Until now, numerous studies on the pathogenicity of *Vibrio* were conducted. According to the current understanding, the pathogenic factors of *Vibrio* include TDH and TRH, type III secretion systems, and adhesion factors. Moreover, when the pathogen achieves an appropriate number of bacterial cell density, the QS mechanism is activated by the expression of these virulence factors. However, the depth and breadth of the current research on the differences between the specific mechanisms of growth, colonization, infection, and pathogenicity of *Vibrio* invading the intestinal environment and the *in vitro* environment are still insufficient. With increasing attention paid to the intestinal microecological environment, especially the role of intestinal microbiota for the immune system, the interaction between *Vibrio* and intestinal microorganisms is undoubtedly an important direction to deepen our understanding of the pathogenicity of *Vibrio*. After *Vibrio* invades the intestinal tract, it may disturb the normal ecological balance of the intestinal tract and may cause intestinal wall damage or dysfunction through pathogenic factors. At the same time, intestinal microbiota tries to prevent the growth of the pathogen in different ways including colonization resistance, competition for nutrients, antibiotic production, and resistance to adhesion of the pathogen to the mucus membrane and enhancing the immunity of the host to the pathogen.

## Author contributions

HS: conceptualization, supervision, and writing—review and editing. CZ: investigation, supervision, and writing—review and editing. XF: writing—review and editing. SK, JW, and ZL: investigation and writing—review. QK: editing. HM: conceptualization, supervision, funding acquisition, and writing—review and editing. FS: conceptualization and writing—review and editing. All authors contributed to the article and approved the submitted version.

## Funding

This work was supported by the National Key Research and Development (R&D) Program of China (2017YFC1600703).

## Conflict of interest

The authors declare that the research was conducted in the absence of any commercial or financial relationships that could be construed as a potential conflict of interest.

## Publisher's note

All claims expressed in this article are solely those of the authors and do not necessarily represent those of their affiliated organizations, or those of the publisher, the editors and the reviewers. Any product that may be evaluated in this article, or claim that may be made by its manufacturer, is not guaranteed or endorsed by the publisher.
